# Foods, macronutrients and breast cancer risk in postmenopausal women: a large UK cohort

**DOI:** 10.1093/ije/dyy238

**Published:** 2018-11-08

**Authors:** Timothy J Key, Angela Balkwill, Kathryn E Bradbury, Gillian K Reeves, Ai Seon Kuan, Rachel F Simpson, Jane Green, Valerie Beral

**Affiliations:** 1 Cancer Epidemiology Unit, Nuffield Department of Population Health, University of Oxford, Oxford, UK; 2 National Institute for Health Innovation, School of Population Health, University of Auckland, Auckland, New Zealand

**Keywords:** Foods, alcohol, macronutrients, breast cancer risk, estrogen receptor status

## Abstract

**Background:**

The role of diet in breast cancer aetiology is unclear; recent studies have suggested associations may differ by estrogen receptor status.

**Methods:**

Baseline diet was assessed in 2000–04 using a validated questionnaire in 691 571 postmenopausal UK women without previous cancer, who had not changed their diet recently. They were followed by record linkage to national cancer and death databases. Cox regression yielded adjusted relative risks for breast cancer for 10 food items and eight macronutrients, subdivided mostly into five categories of baseline intake. Trends in risk across the baseline categories were calculated, assigning re-measured intakes to allow for measurement error and changes in intake over time; *P*-values allowed for multiple testing.

**Results:**

Women aged 59.9 (standard deviation (SD 4.9)) years at baseline were followed for 12 (SD 3) years; 29 005 were diagnosed with invasive breast cancer. Alcohol intake had the strongest association with breast cancer incidence: relative risk (RR) 1.08 [99% confidence interval (CI) 1.05–1.11] per 10 g/day higher intake, *P* = 5.8 × 10^−14^. There were inverse associations with fruit: RR 0.94 (99% CI 0.92–0.97) per 100 g/day higher intake, *P* = 1.1 × 10^−6^, and dietary fibre: RR 0.91 (99% CI 0.87–0.96) per 5 g/day increase, *P* = 1.1 × 10^−4^. Fruit and fibre intakes were correlated (ρ = 0.62) and were greater among women who were not overweight, so residual confounding cannot be excluded. There was no heterogeneity for any association by estrogen receptor status.

**Conclusions:**

By far the strongest association was between alcohol intake and an increased risk of breast cancer. Of the other 17 intakes examined, higher intakes of fruit and fibre were associated with lower risks of breast cancer, but it is unclear whether or not these associations are causal.

**Key Messages**

- In this large prospective study, we systematically examined associations of 10 foods and eight macronutrients with breast cancer risk, overall and by estrogen receptor status.
- The strongest finding was a positive association of alcohol consumption with breast cancer risk, in line with previous evidence.
- None of the 17 other foods and macronutrients examined was strongly associated with breast cancer risk; both fruit and fibre intakes, which are correlated, showed inverse associations with risk, but it is unclear if these associations are causal.
- For none of the dietary items examined was there evidence of heterogeneity by estrogen receptor status.

## Introduction

Breast cancer incidence is generally greater in high-income than low-income countries.^1^^,^^2^ Much of this variation is due to reproductive factors such as parity and breastfeeding, together with variations in screening and diagnosis,^2^^,^^3^ but other risk factors might be involved. Many studies have explored the possible role of diet, with the main hypotheses being that risk may be increased by high intakes of alcohol, fat, meat and dairy products, and that risk may be decreased by high intakes of fibre, fruits and vegetables.^4^ There is now convincing evidence that alcohol increases the risk of breast cancer.^5^ A recent systematic review of prospective studies concluded that there was no definite evidence that any other dietary factors were associated with the risk of developing breast cancer, although there was limited evidence that relatively high intakes of vegetables might decrease the risk of estrogen receptor-negative (ER-ve) breast cancer.^6^ Another recent review concluded that high intakes of red and processed meat intake may be risk factors for breast cancer.^7^

We report here results from an analysis of the relation between intakes of a wide range of foods and macronutrients and breast cancer incidence among postmenopausal women in the Million Women Study, a large prospective study in the UK. To cover the most prominent hypotheses for effects of diet on breast cancer risk,^4^ we examined risk in relation to 18 dietary items and macronutrients (meat type and quantity, fish, milk, cheese, yogurt, eggs, fruit, vegetables, alcohol, energy, percentage of energy from protein, dairy protein, total fat, saturated fat, carbohydrate, free sugars and dietary fibre). Associations were examined overall for any invasive breast cancer, and separately by ER status.

## Methods

### Study population

Participants in the Million Women Study were recruited from women invited to the National Health Service (NHS) Breast Screening Programme in England and Scotland between 1996 and 2001. A total of 1.3 million women aged 56 years [standard deviation (SD) 6] on average joined the study by completing the recruitment questionnaire, which asked about social, demographic and lifestyle factors. Postal re-surveys have been done every 3–5 years and online questionnaires about dietary intake have been administered since 2010. The study was approved by the Oxford and Anglia Multi-Centre Research Ethics Committee, and all women gave written consent. Further details are on the study website.^8^^,^^9^

### Assessment of diet

An average of 3.3 years after recruitment, women were sent a questionnaire which updated information collected at recruitment and collected new information on diet. This 3-year re-survey, which asked for the first time about dietary intakes, was completed in 2000–04 and is the baseline for the analyses reported here. Participants were asked about their usual diet during a typical week, including about 130 quantitative or semi-quantitative questions (see: http://www.millionwomenstudy.org/files/mws-web2.pdf). Nutrient intakes were calculated by multiplying the frequency of consumption of each food by the specified portion size and the nutrient composition of that item. The short-term repeatability of most of the diet questions was good, and in comparison with intakes of nine macronutrients estimated from 7-day food diaries (with portion sizes weighed or assessed by comparison with photographs), the median energy-adjusted correlation coefficient was 0.55 (range from 0.27 for polyunsaturated fat to 0.75 for alcohol).^10^

For these analyses we categorized reported intakes at baseline into five groups except for fish, where three categories were used: no fish; any fatty fish (salmon, sardines, kippers/herring, trout, and mackerel]; and non-fatty fish only [tuna, cod/haddock, ‘fish & chips’ and other seafood). For meat, the five categories were of the type of meat consumed: never ate meat; ate poultry but no red meat (with red meat comprising beef, lamb, pork, beefburger/hamburger, kidney, and liver/paté); consumers of red meat but no processed meat; consumers of red meat and at least one type of processed meat (bacon, ham or sausages); and consumers of red meat and at least two types of processed meat. For other foods the categories were selected to provide substantial numbers in each of the five groups, while respecting the integer nature of some of the data (e.g. items of fruit consumed per day). Vegetables comprised cooked vegetables (except potatoes) and salad items/raw vegetables; and fruit comprised fresh fruit, dried fruit and stewed or tinned fruit. For the eight macronutrients studied here, the cut-points for the five groups were quintiles of intake. In supplementary analyses we examined breast cancer risk: by types of fruit and sources of fibre (for sources of dietary fibre, the mean intakes were from the baseline questionnaire because the coding of food subgroups and composite foods was not directly comparable to that in the 24-h dietary assessment data); by intakes of alcohol, stratified by intakes of fruit and fibre; and by intakes of soya foods and types of vegetables.

Repeat measures of intake in each baseline category were derived from a web-based 24-h dietary assessment tool, the Oxford WebQ,^11^ completed by 19 478 women on average 10 years after baseline and before the end of follow-up.

### Ascertainment of breast cancer

Participants were followed by record linkage to the UK NHS databases on cancer registrations and deaths. Data for England were provided by NHS Digital and the Office for National Statistics, and for Scotland by the Information Services Division, NHS Scotland. Information on estrogen receptor (ER) status of the incident breast tumours was provided by Public Health England/National Cancer Registration and Analysis Service; this information is incomplete, particularly during the early years of follow-up. Overall 1% (*n* = 7647) of the cohort has been lost to follow-up. and individuals contributed person-years until they were lost. Diagnoses were coded to the *International Classification of Diseases, 10th Revision* (ICD-10). The endpoint for this study was incident invasive breast cancer (ICD-10 C50).

### Statistical analysis

The dietary factors examined were consumption of 10 food groups including alcohol, and eight macronutrients or subtypes of macronutrients. Associations between intakes of food groups and macronutrients were examined by calculating Spearman non-parametric correlation coefficients. For analyses of relative risk of breast cancer, women were generally divided into five baseline categories of food or nutrient intake. For estimating trends in risk, the food and nutrient intakes assigned to each baseline category were the means in each baseline category re-measured in the 24-h dietary recall (for women with more than one 24-h dietary recall, data were used just from the first). This non-parametric approach reduces the impact of regression dilution bias and other forms of measurement error.^12^

Woman-years were calculated from the date the baseline dietary questionnaire was completed up to whichever came first: diagnosis of cancer, emigration, death, loss to follow-up or the last date when cancer incidence data were complete. The last date of follow-up was 31 December 2014. Cox regression models with time in study as the underlying time variable were used to estimate rate ratios (RRs) and 99% confidence intervals (99% CIs) of breast cancer by dietary factors. Analyses were stratified by year of birth, region of residence (10 geographical regions in England, and Scotland) and calendar year of completion of the dietary questionnaire, and adjusted for dietary energy intake (fifths), socioeconomic group (fifths, based on the Townsend score^13^), body mass index (<25, 25–29.9, ≥30 kg/m^2^), height (<160, 160–164.9, ≥165 cm), alcohol consumption (except for the analysis of alcohol and risk; none, 1–2, 3–6, 7–14, ≥15 drinks per week), smoking (never, past, current <15, current ≥15 cigarettes per day), age at menarche (≤11, 12–13, 14–15, ≥16 years), strenuous exercise (none, ≤1 per week, >1 per week), parity and age at first birth (parity 0, 1–2, ≥3 cross-classified by age at first birth <25, 25–29, ≥30 years), highest education (none, technical, secondary, tertiary) and ever use of hormonal therapy for menopause (no, yes). The non-dietary data were from the 3-year re-survey, except for socioeconomic group, strenuous exercise and height, which were from the recruitment questionnaire. To ensure that the same women were being compared in all analyses, the small number with a missing value for each particular variable were assigned to a separate category for that variable.

In analyses of factors with more than two categories, RRs were treated as floating absolute risks, allowing estimation of group-specific confidence intervals for all categories including the reference group.^14^ This method allows any two exposure groups to be compared. Most dietary variables considered here have no natural baseline category, so the middle category was used as the reference group. For trends in breast cancer risk per specified increment in dietary intake, conventional CIs are used (for alcohol, the estimates of linear trend were in drinkers only, because non-drinkers may have different characteristics). Heterogeneity in the associations between women with ER+ve and ER-ve breast cancer was assessed with likelihood ratio tests calculated from case-only models in which the outcome was defined as ER+ or ER- disease.

We examined trends in breast cancer risk overall and subdivided by ER status. Many tests for trend and hundreds of relative risks and confidence intervals are presented here, and there is no agreed convention for dealing with this extent of multiple testing. When examining trends in breast cancer risk for the 18 dietary items examined, we used the magnitudes of χ^2^ statistics to assess the strengths of associations and applied a Bonferroni correction to nominal *P*-values by multiplying the values by 18. Some allowance for the hundreds of individual RR estimates in the tables and plots was made by quoting their 99% confidence intervals. In supplementary analyses, we examined risk by subtypes of fruit and sources of fibre, by intakes of alcohol stratified by intakes of fruit and fibre (because it has been suggested that high intakes of dietary fibre may attenuate the increase in risk caused by alcohol^15^) and by intakes of soya foods and types of vegetables. All statistical analyses were performed using Stata statistical software, release 15.1 (StataCorp., College Station, TX).

## Results

The baseline diet questionnaire was completed by 861 918 women. We excluded 47 212 with previous cancer (other than non-melanoma skin cancer) at baseline and 123 099 who reported at baseline that they had changed their diet in the previous 5 years due to illness. Women with energy intakes <2100 kJ/day (d) (500 kcal/d) or more than 14 700 kJ/d (3500 kcal/d) were excluded. After these exclusions, 691 571 women were eligible for these analyses. Table 1 shows their characteristics and average intakes of food groups, alcohol and macronutrients at baseline, together with details of follow-up. Table 2 shows the intakes of foods, alcohol and macronutrients in relation to non-dietary characteristics; intakes of alcohol, fruit and fibre all tended to be greater among women who were taller, were not overweight or obese, had fewer children and were of higher socioeconomic status. Compared with women reporting being in excellent or good health, women in fair or poor health reported markedly lower intakes of fruit, vegetables, alcohol and dietary fibre (Table S1, available as Supplementary data at *IJE* online). Table S2, available as Supplementary data at *IJE* online, shows the correlations between the food groups, alcohol and macronutrients at baseline (the latter expressed as percentage of energy intake), and Table S3, available as Supplementary data at *IJE* online, shows the mean intakes estimated from 24-h dietary assessments 10 years after baseline, which confirm the rankings at baseline.

**Table 1.** Characteristics of 691 571 women at baseline, and details of follow-up

Characteristics	Mean (SD)
Personal	
Age at first dietary assessment, years	59.9 (4.9)
Height, m	1.62 (0.07)
BMI, kg/m^2^	25.9 (4.4)
Number of full-term pregnancies	2.1 (1.2)
Foods and alcohol	
Meat g/d	55.7 (34.0)
Milk g/d	264 (176)
Cheese g/d	17.9 (14.6)
Yogurt g/d	72.8 (68.9)
Eggs g/d	17.1 (13.2)
Fruit g/d	186 (146)
Vegetables g/d	112 (80)
Alcohol g/d	6.6 (8.4)
Macronutrients	
Energy kJ/d	6772 (1802)
Protein % energy	16.4 (2.7)
Dairy protein % energy	4.0 (1.7)
Total fat % energy	33.4 (6.0)
Saturated fat % energy	11.7 (3.6)
Carbohydrate % energy	47.8 (7.0)
Free sugars % energy	12.8 (6.1)
Dietary fibre g/d	13.4 (4.8)
Follow-up for breast cancer	
Person-years of follow-up per woman, mean (SD)	11.9 (3.0)
Incident breast cancers, *n*	29 005
ER+ve breast cancers, *n*	10 838
ER-ve breast cancers, *n*	1658

ER+ve, estrogen receptor-positive breast cancers; ER-ve, estrogen receptor-negative breast cancers; d, day.

**Table 2.** Associations of foods, alcohol and macronutrients with other characteristics at baseline

	Height (cm)		BMI (kg/m^2^)		Full-term pregnancies		HT use		SES (tertile)	
	<165	165+	<25	25+	<3	3+	Not current	Current	Highest	Remainder
*N*	416 846	266 281	303 905	335 363	472 118	217 613	484 088	190 170	229 964	456 522
Foods and alcohol										
Meat (g/d)	55.0	56.9	53.6	58.0	55.9	55.3	55.4	57.1	57.8	54.6
Milk (g/d)	264	266	264	267	263	268	267	259	262	266
Cheese (g/d)	17.1	19.1	19.1	16.9	18.0	17.7	17.9	18.1	18.6	17.6
Yogurt (g/d)	70.9	76.1	70.4	76.3	73.5	71.3	73.9	70.6	76.5	70.9
Eggs (g/d)	16.7	17.8	16.4	17.7	16.8	17.7	17.1	17.2	17.0	17.1
Fruit (g/d)	180	196	192	183	188	180	187	184	198	179
Vegetables (g/d)	108	118	115	112	114	109	112	115	118	109
Alcohol (g/d)	6.3	7.1	7.5	6.1	6.9	6.0	6.3	7.5	7.3	6.3
Nutrients										
Energy kJ/d	6647	6976	6843	6768	6781	6758	6800	6751	6896	6710
Protein % energy	16.4	16.4	16.2	16.6	16.4	16.4	16.4	16.5	16.5	16.4
Dairy protein % energy	4.0	4.0	4.0	4.0	4.0	4.0	4.0	4.0	4.0	4.0
Total fat % energy	33.2	33.6	33.5	33.2	33.3	33.6	33.4	33.3	33.4	33.4
Saturated fat % energy	11.6	11.8	11.8	11.5	11.6	11.8	11.7	11.6	11.6	11.7
Carbohydrate % energy	48.0	47.5	47.8	48.1	47.8	48.0	47.9	47.5	47.5	48.0
Free sugars % energy	12.8	12.8	12.7	12.9	12.7	13.0	12.9	12.6	12.6	12.9
Dietary fibre (g/d)	13.1	13.9	13.8	13.3	13.5	13.1	13.5	13.4	13.9	13.1

BMI, body mass index; HT, hormonal therapy for menopause; SES, socioeconomic status; d, day.

**Figure 1.**
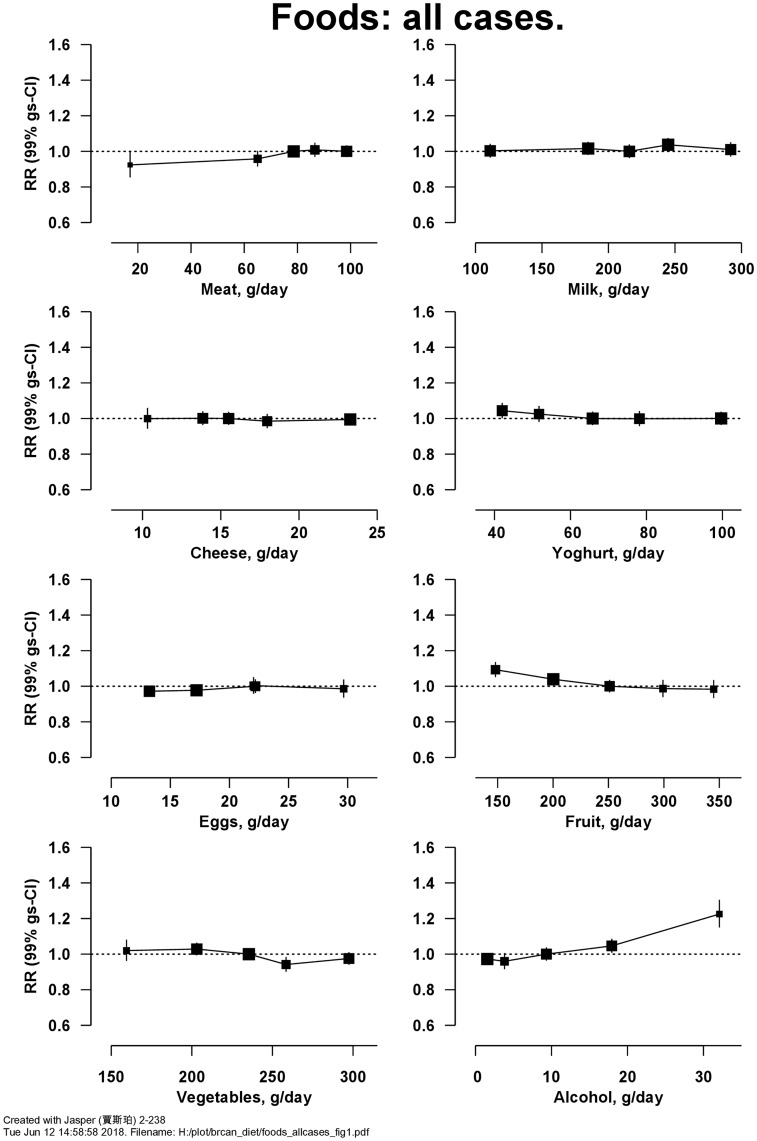
Relative risk of breast cancer in Million Women Study participants by intake of foods and alcohol. Risks are stratified by region, with attained age as the underlying time variable and adjusted for socioeconomic status, body mass index, height, smoking, current use of hormonal therapy for menopause, dietary energy intake and alcohol consumption (except for the analysis of alcohol and risk). Relative risks (RRs) are represented by squares (with their 99% confidence intervals as lines), each with area inversely proportional to the variance of the log RR, thereby indicating the amount of statistical information for that particular RR.

**Figure 2.**
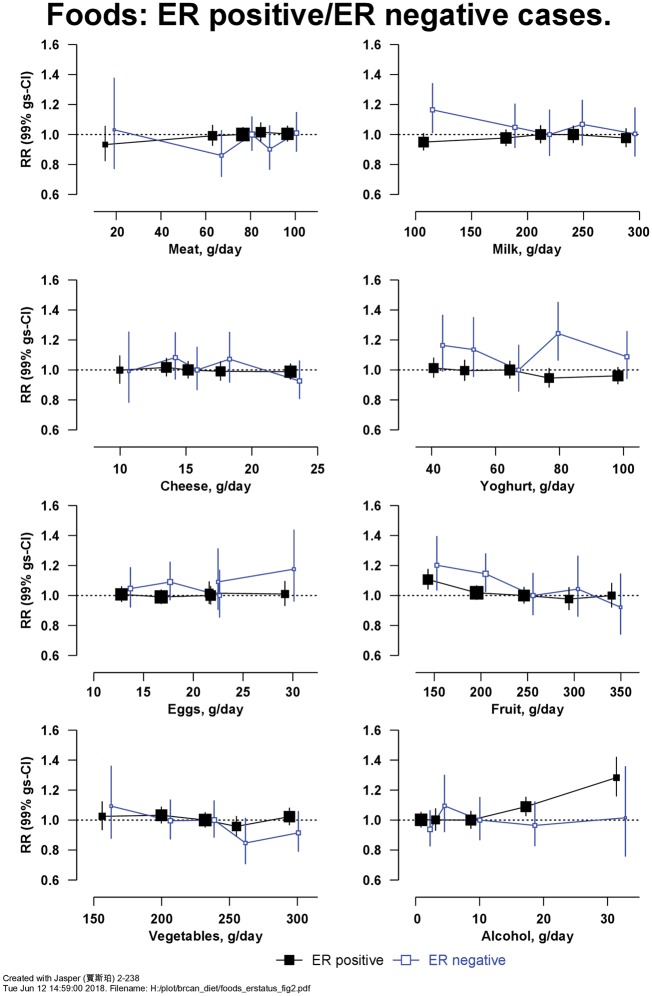
Relative risk of breast cancer in Million Women Study participants by intake of foods and alcohol by estrogen receptor (ER) status. Risks are stratified by region, with attained age as the underlying time variable and adjusted for socioeconomic status, body mass index, height, smoking, current use of hormonal therapy for menopause, dietary energy intake and alcohol consumption (except for the analysis of alcohol and risk). Relative risks (RRs) are represented by squares (with their 99% confidence intervals as lines), each with area inversely proportional to the variance of the log RR, thereby indicating the amount of statistical information for that particular RR.

After 12 (SD 3) years of follow-up, 29 005 women were diagnosed with incident invasive breast cancer, among whom information on ER status was available for 10 838 ER+ve and 1658 ER-ve breast cancers. Figure 1 shows the RRs for breast cancer in relation to the intakes of eight food items. There was a positive association with alcohol, with an RR of 1.08 (99% CI 1.05–1.11, Χ_1_^2^ for trend = 62.1; *P* = 5.8 × 10^−14^) per 10 g/d higher intake (trend in drinkers only). There was also an inverse association of risk with fruit intake, with a RR of 0.94 (99% CI 0.92–0.97, Χ_1_^2^ for trend = 29.4; *P* = 1.1 × 10^−6^) per 100 g/d higher intake (further results for Figure 1 are in Table S4, available as Supplementary data at *IJE* online). Figure 2 shows the associations of intakes of these seven food groups and alcohol separately for ER+ve and ER-ve breast cancer; there was no significant heterogeneity in associations by ER status (Table S4, available as Supplementary data at *IJE* online). Not all women had information available on ER status, but findings in these women did not differ materially from those with available information (results not shown). Breast cancer risk was not associated with the types of meat and fish consumed (Table 3). Breast cancer risk was inversely associated with intake of fresh fruit [RR of 0.95 (99% CI 0.92–0.98) per 100 g/d higher intake, Χ_1_^2^ for trend = 19.5] and dried fruit [RR of 0.95 (99% CI 0.90–1.00) per 10 g/d higher intake, Χ_1_^2^ for trend = 7.10], but not with tinned/stewed fruit or fruit juice (Table S5, available as Supplementary data at *IJE* online). After allowing for multiple testing, breast cancer risk was not associated with consumption of soya foods or types of vegetables (Tables S6 and S7, available as Supplementary data at *IJE* online).

**Table 3.** Associations of intake of type of meat and fish with breast cancer risk

							Cases subdivided by ER status			
Food or food group	Usual intake g/d (mean of intakes from 24-h recalls)				All cases		ER+ve		ER-ve	
					Cases	RR (99% gs-CI)	Cases	RR (99% gs-CI)	Cases	RR (99% gs-CI)
	Poultry	Red meat	Processed meat	Total meat						
Type of meat										
None	5.1	2.9	1.9	10.0	775	0.92 (0.84–1.01)	285	0.86 (0.74–1.00)	50	1.16 (0.80–1.67)
Poultry only	32.3	23.7	10.0	67.8	2830	1.00 (0.95–1.05)	1101	1.00 (0.92–1.08)	145	1.00 (0.80–1.24)
Red meat	32.0	39.3	11.9	85.3	6279	1.02 (0.99–1.06)	2427	0.99 (0.94–1.04)	345	1.11 (0.96–1.27)
Processed (lower)	30.9	38.4	15.1	86.9	10 430	1.02 (1.00–1.05)	3848	0.97 (0.93–1.01)	599	1.20 (1.08–1.33)
Processed (higher)	29.6	42.5	19.0	93.6	8498	1.04 (1.01–1.07)	3105	0.98 (0.93–1.03)	509	1.30 (1.15–1.46)
					Χ_4_^2^ for heterogeneity = 12.62			Χ_4_^2^ for heterogeneity by ER status = 10.61		
	White fish	Oily fish	Seafood	Total fish						
Type of fish										
None	4.0	2.5	1.0	6.6	755	0.93 (0.85–1.02)	274	0.90 (0.77–1.05)	42	0.88 (0.59–1.31)
White only	15.2	9.4	2.7	24.6	11 957	1.00 (0.98–1.02)	4460	1.00 (0.96–1.04)	704	1.00 (0.91–1.10)
Some oily	16.8	16.4	4.0	33.2	15 140	0.98 (0.96–1.00)	5678	1.00 (0.96–1.04)	856	0.98 (0.89–1.07)
					Χ_2_^2^ for heterogeneity = 5.31			Χ_2_^2^ for heterogeneity by ER status = 0.24		

ER+ve, estrogen receptor-positive breast cancers; ER-ve, estrogen receptor-negative breast cancers; gs-CI, group-specific confidence intervals; d, day.

**Figure 3.**
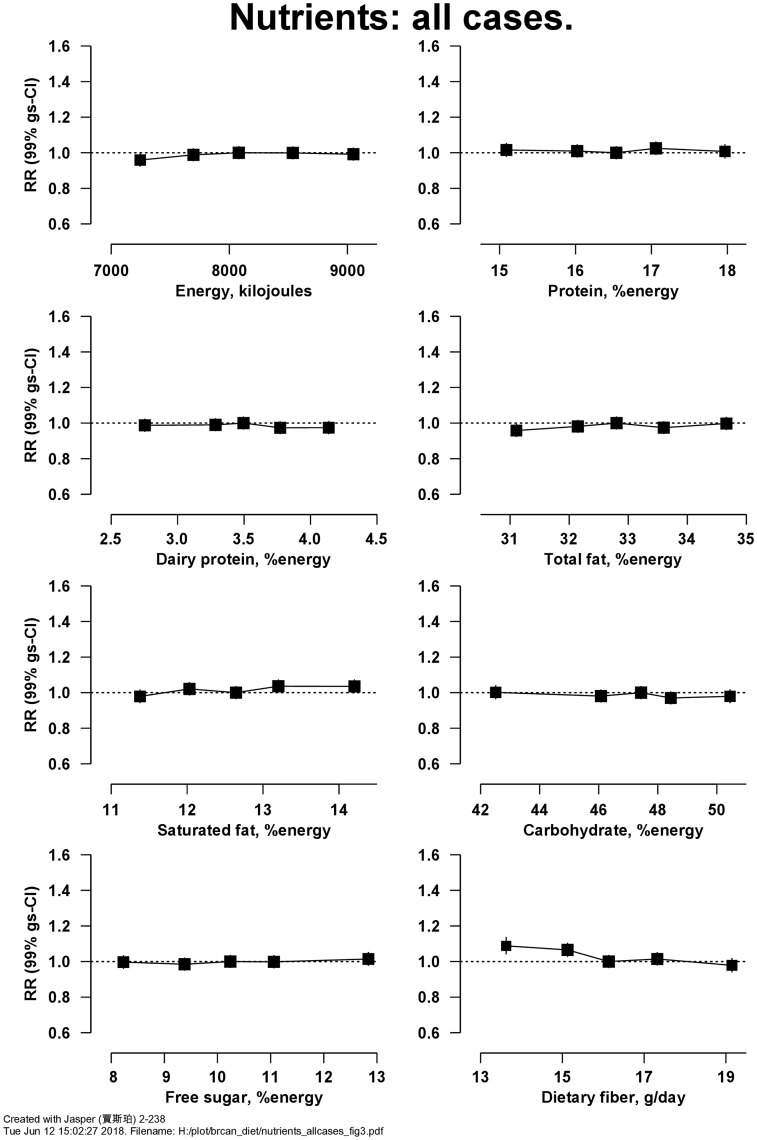
Relative risk of breast cancer in Million Women Study participants by intake of macronutrients. Risks are stratified by region, with attained age as the underlying time variable and adjusted for socioeconomic status, body mass index, height, smoking, current use of hormonal therapy for menopause, dietary energy intake and alcohol consumption. Relative risks (RRs) are represented by squares (with their 99% confidence intervals as lines), each with area inversely proportional to the variance of the log RR, thereby indicating the amount of statistical information for that particular RR.

**Figure 4.**
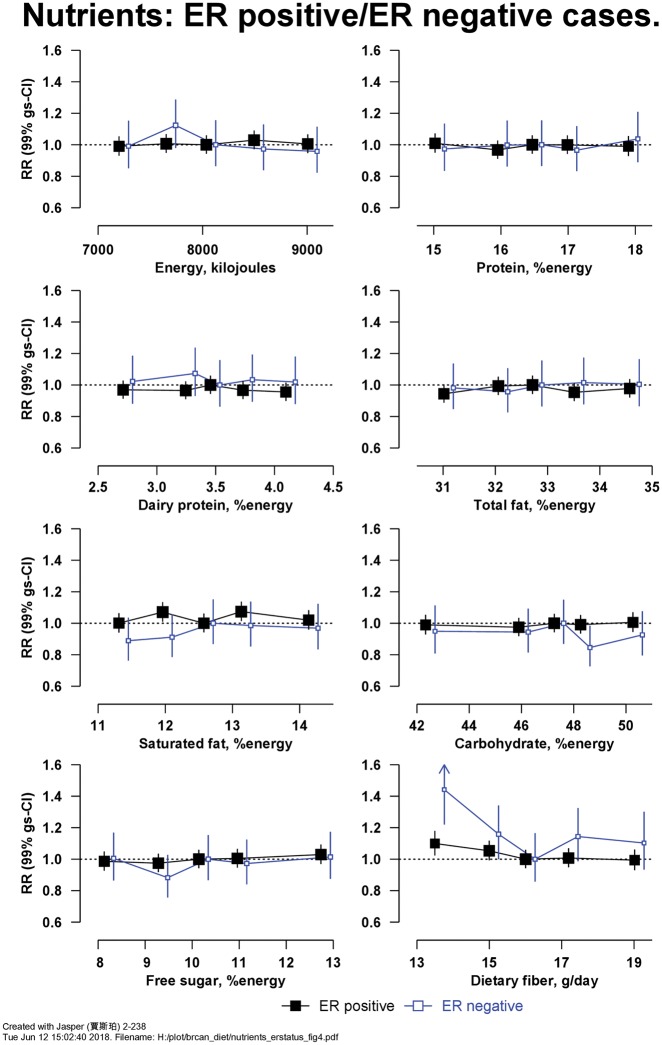
Relative risk of breast cancer in Million Women Study participants by intake of macronutrients by estrogen receptor (ER) status. Risks are stratified by region, with attained age as the underlying time variable and adjusted for socioeconomic status, body mass index, height, smoking, current use of hormonal therapy for menopause, dietary energy intake and alcohol consumption. Relative risks (RRs) are represented by squares (with their 99% confidence intervals as lines), each with area inversely proportional to the variance of the log RR, thereby indicating the amount of statistical information for that particular RR.

Figure 3 shows RRs for breast cancer in relation to intakes of eight macronutrients. There was an inverse association of risk with intake of dietary fibre, with RR = 0.91 (99% CI 0.87–0.96, Χ_1_^2^ for trend = 20.5; *P* = 1.1 × 10^−4^) per 5 g/d higher intake (detailed results for Figure 3 are in Table S8, available as Supplementary data at *IJE* online). Figure 4 shows the associations of intake of these eight macronutrients with risk of ER+ve and ER-ve breast cancer; there was no heterogeneity in associations by ER status (Table S8, available as Supplementary data at *IJE* online); in women without information on ER status, results were similar (results not shown). Breast cancer risk was inversely associated with the intake of fibre from fruit but not from other sources (Table S9, available as Supplementary data at *IJE* online).

Associations of fruit and fibre intake with breast cancer risk were found separately at five levels of alcohol consumption, and associations of alcohol intake with breast cancer risk were similar at three levels of intake of fruit and at five levels of intake of dietary fibre (Table S10, available as Supplementary data at *IJE* online).

## Discussion

We report the results from systematic analyses in a large prospective study of the associations between intakes of 18 dietary factors and the incidence of breast cancer. The positive association with alcohol consumption (*P* = 5.8 × 10^−14^) is expected, given that alcohol is an established cause of breast cancer.^5^ The other statistically significant associations, inverse relationships with fruit and dietary fibre intakes, were considerably weaker than for alcohol (*P* = 1.1 × 10^−6^ and 1.1 × 10^−4^, respectively, after correction for multiple testing). Intakes of fruit and dietary fibre were strongly correlated with each other (correlation coefficient 0.62), therefore the associations of these two dietary items with breast cancer risk are not independent. In a recent meta-analysis,^6^ the intakes of fruit and dietary fibre were also both associated with a small reduction in breast cancer risk; whereas these small associations might be causal, it is also possible that they are largely or wholly due to residual confounding.

In our data, examination of whether there was heterogeneity in dietary associations between ER+ve and ER-ve breast cancers showed no clear evidence for such heterogeneity. Previous studies found no heterogeneity by ER status in the associations of alcohol^16^ or fruit^6^^,^^17–19^ with breast cancer risk, similar to our results. Previous studies suggested that vegetable intakes were inversely associated with risk for ER-ve but not ER+ve breast cancer^6^^,^^16^^,^^19^; but we found no significant association of breast cancer risk with intake of vegetables, and no heterogeneity by ER status. For dietary fibre, results by ER status from previous studies are inconsistent.^20–24^ Overall, the currently available evidence shows no consistent differences in associations of dietary factors with breast cancer risk by ER status, but more data are required, especially for ER-ve disease.

Chhim et al.^15^ suggested that a high intake of dietary fibre might attenuate the adverse effect of alcohol on breast cancer risk, and a subsequent examination of this hypothesis in the EPIC study showed some evidence of an interaction between the associations of alcohol and fibre with risk, with a higher relative risk for breast cancer associated with alcohol in women with a low fibre intake^25^; however, our results do not support this hypothesis.

With 29 000 incident breast cancers in a cohort of almost 700 000 women, this is as far as we are aware the largest single prospective study of diet and breast cancer risk. Nutritional epidemiology may be subject to selective reporting biases,^26^ and therefore we used a systematic approach, assessing the relationships of breast cancer risk with 18 pre-specified dietary factors and allowing for multiple testing in the interpretation of the findings. Other strengths of this study include the virtually complete follow-up for cancer over 12 years, the comprehensive assessment of confounders and the use of re-measured intakes to allow for changes in diet over time and measurement error. Diet at baseline was assessed with a questionnaire which has been validated with reference to 7-day food diaries^11^ and shown to predict risks of other conditions.^27^ Tests for trend across groups categorised by their baseline intakes of foods and nutrients mean re-measured intakes for each category from 24-h dietary assessments a decade later, to provide estimates of usual dietary intake within each baseline category.^11^^,^^12^ The re-measured dietary intakes confirmed the rankings of categories of intake at baseline, generally with substantial narrowing in the ranges of intakes across categories, as would be expected from regression to the mean due to measurement error and changes over time.^28^

The study has some limitations. Like other observational studies, the results may be influenced by residual confounding and reverse causation. Also, for some dietary factors in this population, such as for energy from protein, dairy protein and fat, the range of re-measured intakes between the lowest and highest intakes at baseline was so narrow that it limits the power to detect associations with these factors. The study was also conducted among predominantly White women in the UK.

This study showed a strong positive association of alcohol consumption with breast cancer risk, in line with previous studies. None of the other foods and nutrients examined was strongly associated with breast cancer risk; both fruit and fibre showed modest inverse associations with risk, but it is unclear if these associations are causal.

## Funding

This work was supported by Cancer Research UK (grant no. C570/A16491), the UK Medical Research Council (grant no. MR/K02700X/1) and the Wellcome Trust, Our Planet Our Health (Livestock, Environment and People—LEAP, award number 205212/Z/16/Z). K.E.B. is supported by the Girdlers' New Zealand Health Research Council Fellowship. The funders had no role in study design, data collection and analysis, decision to publish or preparation of the manuscript. The authors had full access to the data and analyses, and were solely responsible for the decision to submit for publication.

## Supplementary Material

Supplementary Data
