# Erratum: Foods, macronutrients and breast cancer risk in postmenopausal women: a large UK cohort

**DOI:** 10.1093/ije/dyy281

**Published:** 2018-12-05

**Authors:** Timothy J Key, Angela Balkwill, Kathryn E Bradbury, Gillian K Reeves, Ai Seon Kuan, Rachel F Simpson, Jane Green, Valerie Beral

First published online: 8 November 2018, Int J Epidemiol, doi: https://doi.org/10.1093/ije/dyy238

In the originally published version of this article, the first and last names of the authors Angela Balkwill and Ai Seon Kuan appeared in the wrong order. This has now been corrected.

