# Supplementary material for: Foods, macronutrients and breast cancer risk in postmenopausal women: a large UK cohort
Source: Int J Epidemiol. 2018 Nov 8;48(2):489–500. doi: 10.1093/ije/dyy238 (PMC6469308; doi:10.1093/ije/dyy238)
Supplement: Supplementary Data [file dyy238_supplementary_data.docx]

**Foods, macronutrients and breast cancer risk: a large UK cohort**

Timothy J Key, Angela Balkwill, Kathryn E Bradbury, Gillian K Reeves, Ai Seon Kuan, Rachel F Simpson, Jane Green, Valerie Beral

**Supplementary Tables**

**Table S1.** Food, alcohol and macronutrient intakes^a^ in relation to self-rated health of participants at baseline

| **Food or macronutrient** | **Excellent/good health, %** | **Fair/poor health, %** |
| --- | --- | --- |
|  |  |  |
| **Foods and alcohol** |  |  |
| *Type of meat* |  |  |
| None | 3.2 | 2.9 |
| Poultry only | 10.3 | 9.3 |
| Red meat | 22.0 | 20.4 |
| Processed (lower) | 36.1 | 35.6 |
| Processed (higher) | 28.5 | 31.8 |
| *Meat, portions per week* |  |  |
| 0 | 4.8 | 4.7 |
| 1-2 | 15.0 | 17.4 |
| 3-4 | 31.7 | 33.1 |
| 5 | 17.7 | 16.4 |
| 6+ | 30.9 | 28.5 |
| *Type of fish* |  |  |
| None | 2.8 | 3.9 |
| Some fatty | 56.1 | 48.6 |
| White only | 41.1 | 47.5 |
| *Milk, fifth of intake* |  |  |
| 1 | 20.3 | 19.7 |
| 2 | 21.6 | 19.9 |
| 3 | 18.8 | 18.1 |
| 4 | 21.0 | 20.9 |
| 5 | 18.3 | 21.4 |
| *Eggs, per week* |  |  |
| ≤1 | 28.4 | 28.8 |
| 2 | 30.4 | 30.5 |
| 3 | 18.8 | 18.0 |
| 4 | 12.4 | 12.4 |
| 5+ | 10.0 | 10.4 |
| *Fruit, portions per day* |  |  |
| <1 | 18.9 | 29.3 |
| 1- | 31.9 | 33.1 |
| 2- | 24.8 | 20.2 |
| 3- | 12.7 | 9.2 |
| 4+ | 11.7 | 8.3 |
| *Vegetables, tablespoons per day* |  |  |
| <1 | 7.6 | 15.2 |
| 1- | 24.1 | 28.9 |
| 2- | 28.0 | 25.2 |
| 3- | 15.7 | 12.5 |
| 4+ | 24.5 | 18.3 |
| *Cheese, times per week* |  |  |
| 0 | 8.5 | 9.3 |
| 1 | 21.1 | 23.2 |
| 2 | 22.3 | 23.7 |
| 3 | 17.6 | 17.3 |
| 4+ | 30.5 | 26.4 |
| *Yogurt, times per week* |  |  |
| 0 | 18.7 | 22.0 |
| 1-2 | 15.7 | 15.9 |
| 3-4 | 22.6 | 23.0 |
| 5-6 | 18.0 | 15.8 |
| 7+ | 25.1 | 23.5 |
| *Alcohol, drinks per week* |  |  |
| 0 | 32.2 | 49.2 |
| 1-2 | 14.2 | 13.0 |
| 3-6 | 23.7 | 18.1 |
| 7-14 | 23.6 | 15.4 |
| 15+ | 6.3 | 4.3 |
|  |  |  |
| **Macronutrients** |  |  |
| *Energy, kJ per d: quintiles* |  |  |
| 1 | 18.7 | 24.4 |
| 2 | 20.0 | 19.9 |
| 3 | 20.4 | 18.5 |
| 4 | 20.5 | 18.1 |
| 5 | 20.4 | 19.1 |

| **Food or macronutrient** | **Excellent/good health, %** | **Fair/poor health, %** |
| --- | --- | --- |
| *Protein, % energy: quintiles* |  |  |
| 1 | 18.9 | 23.9 |
| 2 | 20.0 | 20.0 |
| 3 | 20.3 | 19.0 |
| 4 | 20.5 | 18.3 |
| 5 | 20.3 | 18.8 |
| *Dairy protein, % energy: quintiles* |  |  |
| 1 | 19.6 | 21.5 |
| 2 | 20.2 | 19.3 |
| 3 | 20.3 | 18.8 |
| 4 | 20.2 | 19.3 |
| 5 | 19.7 | 21.1 |
| *Total fat, % energy: quintiles* |  |  |
| 1 | 19.9 | 19.6 |
| 2 | 20.0 | 19.8 |
| 3 | 20.0 | 19.8 |
| 4 | 20.1 | 20.0 |
| 5 | 20.0 | 20.7 |
| *Saturated fat, % energy: quintiles* |  |  |
| 1 | 20.3 | 18.4 |
| 2 | 20.1 | 19.6 |
| 3 | 20.0 | 20.0 |
| 4 | 19.9 | 20.4 |
| 5 | 19.7 | 21.6 |
| *Carbohydrate, % energy: quintiles* |  |  |
| 1 | 20.8 | 17.5 |
| 2 | 20.5 | 18.4 |
| 3 | 20.1 | 19.6 |
| 4 | 19.7 | 21.0 |
| 5 | 18.9 | 23.5 |
| *Free sugar, % energy: quintiles* |  |  |
| 1 | 20.2 | 19.4 |
| 2 | 20.7 | 17.8 |
| 3 | 20.5 | 18.2 |
| 4 | 20.0 | 19.7 |
| 5 | 18.6 | 24.9 |
| *Dietary fiber, g per day: quintiles* |  |  |
| 1 | 17.9 | 28.2 |
| 2 | 19.6 | 21.5 |
| 3 | 20.4 | 18.6 |
| 4 | 20.9 | 16.5 |
| 5 | 21.3 | 15.2 |

^a^Intake categories from baseline questionnaire

**Table S2.** Correlations between categories of intake of foods, alcohol and macronutrients at baseline^a^

|  | **Meat** | **Milk** | **Cheese** | **Yogurt** | **Eggs** | **Fruit** | **Vegetables** | **Alcohol** | **Energy** | **Protein** | **Dairy protein** | **Total fat** | **Saturated fat** | **Carbohydrate** | **Free sugars** | **Dietary fiber** |
| --- | --- | --- | --- | --- | --- | --- | --- | --- | --- | --- | --- | --- | --- | --- | --- | --- |
| **Meat** | 1.00 |  |  |  |  |  |  |  |  |  |  |  |  |  |  |  |
| **Milk** | 0.07 | 1.00 |  |  |  |  |  |  |  |  |  |  |  |  |  |  |
| **Cheese** | 0.04 | 0.01 | 1.00 |  |  |  |  |  |  |  |  |  |  |  |  |  |
| **Yogurt** | -0.05 | 0.06 | 0.00 | 1.00 |  |  |  |  |  |  |  |  |  |  |  |  |
| **Eggs** | 0.10 | 0.02 | 0.19 | 0.00 | 1.00 |  |  |  |  |  |  |  |  |  |  |  |
| **Fruit** | -0.04 | 0.00 | 0.02 | 0.21 | 0.00 | 1.00 |  |  |  |  |  |  |  |  |  |  |
| **Vegetables** | 0.11 | -0.04 | 0.05 | 0.07 | 0.07 | 0.35 | 1.00 |  |  |  |  |  |  |  |  |  |
| **Alcohol** | 0.08 | -0.14 | 0.09 | -0.06 | 0.05 | 0.00 | 0.13 | 1.00 |  |  |  |  |  |  |  |  |
| **Energy** | 0.35 | 0.27 | 0.31 | 0.15 | 0.25 | 0.21 | 0.24 | 0.08 | 1.00 |  |  |  |  |  |  |  |
| **Protein** | 0.35 | 0.17 | -0.03 | 0.07 | -0.01 | 0.00 | 0.10 | -0.05 | -0.23 | 1.00 |  |  |  |  |  |  |
| **Dairy protein** | -0.18 | 0.54 | 0.23 | 0.39 | -0.05 | 0.00 | -0.11 | -0.15 | -0.07 | 0.28 | 1.00 |  |  |  |  |  |
| **Total fat** | 0.25 | -0.11 | 0.34 | -0.22 | 0.23 | -0.20 | 0.04 | 0.00 | 0.26 | -0.08 | -0.17 | 1.00 |  |  |  |  |
| **Saturated fat** | 0.13 | 0.03 | 0.46 | -0.22 | 0.21 | -0.23 | -0.10 | -0.03 | 0.26 | -0.20 | 0.01 | 0.74 | 1.00 |  |  |  |
| **Carbohydrate** | -0.35 | 0.13 | -0.29 | 0.22 | -0.19 | 0.21 | -0.11 | -0.39 | -0.10 | -0.24 | 0.13 | -0.75 | -0.51 | 1.00 |  |  |
| **Free sugars** | -0.11 | 0.11 | -0.05 | 0.30 | -0.01 | -0.09 | -0.15 | -0.19 | 0.19 | -0.34 | 0.17 | -0.28 | -0.08 | 0.49 | 1.00 |  |
| **Dietary fiber** | 0.08 | 0.12 | 0.09 | 0.17 | 0.06 | 0.62 | 0.49 | 0.00 | 0.51 | -0.03 | -0.07 | -0.09 | -0.15 | 0.14 | -0.09 | 1.00 |

^a^Spearman rank correlations; categories as shown in Supplementary Table S3

**Table S3.** Food, alcohol and macronutrient intakes at baseline and 10 years later

| **Food or macronutrient and categories of intake at baseline** | | | **Mean intake at baseline, g per day** | **Mean intake at 10 years, g per day^a^** |
| --- | --- | --- | --- | --- |
|  | | |  |  |
| **Foods and alcohol** | | |  |  |
| Meat, portions per week | | |  |  |
|  | | 0 | 0.0 | 17.1 |
|  | | 1-2 | 21.5 | 65.0 |
|  | | 3-4 | 44.9 | 78.6 |
|  | | 5 | 63.4 | 86.6 |
|  | | 6+ | 94.8 | 98.5 |
| Milk, fifths of intake | | |  |  |
|  | | 1 | 72 | 111 |
|  | | 2 | 183 | 185 |
|  | | 3 | 252 | 216 |
|  | | 4 | 326 | 245 |
|  | | 5 | 505 | 292 |
| Cheese, portions per week | | |  |  |
|  | | 0 | 0.0 | 10.3 |
|  | | 1 | 7.1 | 13.9 |
|  | | 2 | 14.3 | 15.5 |
|  | | 3 | 21.4 | 18.0 |
|  | | 4+ | 37.2 | 23.3 |
| Yogurt, portions per week | | |  |  |
|  | | 0 | 0.0 | 42.0 |
|  | | 1-2 | 36.3 | 51.7 |
|  | | 3-4 | 77.0 | 65.8 |
|  | | 5-6 | 115.2 | 78.1 |
|  | | 7+ | 171.4 | 99.7 |
| Eggs, portions per week | | |  |  |
|  | | ≤1 | 3.9 | 13.2 |
|  | | 2 | 14.3 | 17.2 |
|  | | 3 | 21.4 | 22.2 |
|  | | 4 | 28.6 | 22.0 |
|  | | 5+ | 45.4 | 29.7 |
| Fruit, portions per day | | |  |  |
|  | | 0-0.99 | 51 | 148 |
|  | | 1-1.99 | 130 | 200 |
|  | | 2-2.99 | 225 | 251 |
|  | | 3-3.99 | 312 | 299 |
|  | | 4+ | 450 | 345 |
| Vegetables, tablespoons per day^b^ | | |  |  |
|  | | <1 | 25 | 159 |
|  | | 1-1.99 | 57 | 203 |
|  | | 2-2.99 | 97 | 235 |
|  | | 3-3.99 | 135 | 258 |
|  | | 4.00+ | 225 | 297 |
| Alcohol, drinks per week | | |  |  |
|  | | 0 | 0.0 | 1.6 |
|  | | 1-2 | 2.3 | 3.8 |
|  | | 3-6 | 6.2 | 9.3 |
|  | | 7-14 | 14.1 | 17.9 |
|  | | 15+ | 29.7 | 32.1 |
|  | | |  |  |
| **Macronutrients** | | |  |  |
| Energy, fifths, kJ per day | | |  |  |
|  | 1 | | 4362 | 7246 |
|  | 2 | | 5790 | 7697 |
|  | 3 | | 6689 | 8081 |
|  | 4 | | 7633 | 8538 |
|  | 5 | | 9385 | 9051 |
| Protein, fifths, % energy | | |  |  |
|  | 1 | | 12.9 | 14.9 |
|  | 2 | | 15.0 | 15.8 |
|  | 3 | | 16.3 | 16.5 |
|  | 4 | | 17.6 | 16.9 |
|  | 5 | | 20.2 | 17.8 |
| Dairy protein, fifths, % energy | | |  |  |
|  | 1 | | 1.9 | 2.8 |
|  | 2 | | 3.1 | 3.3 |
|  | 3 | | 3.8 | 3.5 |
|  | 4 | | 4.7 | 3.8 |
|  | 5 | | 6.5 | 4.1 |
| Total fat, fifths, % energy | | |  |  |
|  | 1 | | 25.0 | 30.4 |
|  | 2 | | 30.3 | 31.6 |
|  | 3 | | 33.4 | 32.8 |
|  | 4 | | 36.4 | 33.7 |
|  | 5 | | 41.8 | 35.3 |
| Saturated fat, fifths, % energy | | |  |  |
|  | 1 | | 7.2 | 11.3 |
|  | 2 | | 9.6 | 12.0 |
|  | 3 | | 11.3 | 12.5 |
|  | 4 | | 13.2 | 13.3 |
|  | 5 | | 17.2 | 14.2 |
| Carbohydrate, fifths,% energy | | |  |  |
|  | 1 | | 38.0 | 42.4 |
|  | 2 | | 44.5 | 46.0 |
|  | 3 | | 47.9 | 47.4 |
|  | 4 | | 51.4 | 48.4 |
|  | 5 | | 57.4 | 50.4 |
| Free sugars, fifths, % energy | | |  |  |
|  | 1 | | 5.2 | 8.2 |
|  | 2 | | 9.4 | 9.4 |
|  | 3 | | 12.2 | 10.2 |
|  | 4 | | 15.3 | 11.1 |
|  | 5 | | 21.9 | 12.8 |
| Dietary fiber, fifths, g per day | | |  |  |
|  | 1 | | 7.5 | 13.6 |
|  | 2 | | 10.6 | 15.1 |
|  | 3 | | 13.1 | 16.1 |
|  | 4 | | 15.7 | 17.3 |
|  | 5 | | 20.4 | 19.1 |

^a^Mean values from 24-hour dietary assessments

^b^Level of detail in questions on vegetables on baseline questionnaire and 24-hour dietary assessment differed substantially

**Table S4.** Associations of intake of foods and alcohol with breast cancer risk

|  |  |  | |  | **Cases subdivided by ER status** | | | | |
| --- | --- | --- | --- | --- | --- | --- | --- | --- | --- |
|  |  | **All cases** | |  | **ER+ve** | |  | **ER-ve** | |
| **Food or food group** | **Usual intake g/d (mean)**^a^ | **Cases** | **RR (99% gs-CI)** |  | **Cases** | **RR (99% gs-CI)** |  | **Cases** | **RR (99% gs-CI)** |
| Meat, portions per week |  |  |  |  |  |  |  |  |  |
| 0 | 17.1 | 1,203 | 0.92 (0.86-1.00) |  | 456 | 0.93 (0.83-1.05) |  | 82 | 1.03 (0.77-1.38) |
| 1-2 | 65 | 4,096 | 0.96 (0.92-1.00) |  | 1,641 | 0.99 (0.93-1.06) |  | 234 | 0.86 (0.72-1.03) |
| 3-4 | 78.6 | 9,006 | 1.00 (0.97-1.03) |  | 3,344 | 1.00 (0.96-1.05) |  | 540 | 1.00 (0.90-1.12) |
| 5 | 86.6 | 5,043 | 1.01 (0.97-1.05) |  | 1,872 | 1.02 (0.96-1.08) |  | 260 | 0.90 (0.77-1.06) |
| 6+ | 98.5 | 8,843 | 1.00 (0.97-1.03) |  | 3,223 | 1.00 (0.96-1.06) |  | 488 | 1.01 (0.89-1.15) |
| Trend per 100g/d^b^ |  |  | *1.11 (1.01-1.22)* |  |  | *1.09 (0.94-1.26)* |  |  | *1.05 (0.72-1.53)* |
|  |  |  |  |  |  |  |  |  |  |
|  |  | *Χ_1_^2^ for trend = 8.93* | |  | *Χ_1_^2^ for heterogeneity by ER status = 0.00* | | | | |
|  |  |  |  |  |  |  |  |  |  |
| Milk, fifths of intake |  |  |  |  |  | | | | |
| 1 | 111 | 5,764 | 1.00 (0.97-1.04) |  | 2,202 | 0.95 (0.90-1.01) |  | 374 | 1.16 (1.01-1.34) |
| 2 | 185 | 6,212 | 1.02 (0.98-1.05) |  | 2,334 | 0.98 (0.93-1.03) |  | 354 | 1.05 (0.91-1.20) |
| 3 | 216 | 5,362 | 1.00 (0.97-1.04) |  | 2,056 | 1.00 (0.94-1.06) |  | 292 | 1.00 (0.86-1.16) |
| 4 | 245 | 6,219 | 1.04 (1.00-1.07) |  | 2,272 | 1.00 (0.95-1.06) |  | 349 | 1.07 (0.93-1.23) |
| 5 | 292 | 5,448 | 1.01 (0.97-1.05) |  | 1,974 | 0.98 (0.92-1.04) |  | 289 | 1.00 (0.86-1.18) |
| Trend per 100g/d^b^ |  |  | *1.01 (0.98-1.04)* |  |  | *1.02 (0.98-1.07)* |  |  | *0.93 (0.83-1.04)* |
|  |  |  |  |  |  |  |  |  |  |
|  |  | *Χ_1_^2^ for trend = 0.63* | |  | *Χ_1_^2^ for heterogeneity by ER status = 2.24* | | | | |
|  |  |  |  |  |  |  |  |  |  |
| Cheese, portions per week |  |  |  |  |  |  |  |  |  |
| 0 | 10.3 | 2,246 | 1.00 (0.95-1.06) |  | 823 | 1.00 (0.91-1.09) |  | 125 | 0.99 (0.78-1.25) |
| 1 | 13.9 | 5,624 | 1.00 (0.97-1.04) |  | 2,113 | 1.02 (0.96-1.08) |  | 344 | 1.08 (0.94-1.25) |
| 2 | 15.5 | 5,947 | 1.00 (0.97-1.03) |  | 2,220 | 1.00 (0.95-1.06) |  | 336 | 1.00 (0.87-1.15) |
| 3 | 18 | 4,619 | 0.98 (0.95-1.02) |  | 1,734 | 0.99 (0.93-1.05) |  | 281 | 1.07 (0.92-1.25) |
| 4+ | 23.3 | 8,058 | 0.99 (0.96-1.03) |  | 3,022 | 0.99 (0.94-1.04) |  | 413 | 0.93 (0.81-1.06) |
| Trend per 10 g/d^b^ |  |  | *0.99 (0.96-1.03)* |  |  | *0.98 (0.92-1.05)* |  |  | *0.91 (0.77-1.07)* |
|  |  |  |  |  |  |  |  |  |  |
|  |  | *Χ_1_^2^ for trend = 0.16* | |  | *Χ_1_^2^ for heterogeneity by ER status = 0.97* | | | | |
|  |  |  |  |  |  |  |  |  |  |
| Yogurt, portions per week |  |  |  |  |  |  |  |  |  |
| 0 | 42 | 4,836 | 1.04 (1.00-1.08) |  | 1,807 | 1.01 (0.95-1.08) |  | 278 | 1.16 (0.99-1.36) |
| 1-2 | 51.7 | 3,968 | 1.02 (0.98-1.07) |  | 1,480 | 1.00 (0.93-1.06) |  | 227 | 1.14 (0.96-1.35) |
| 3-4 | 65.8 | 5,515 | 1.00 (0.97-1.04) |  | 2,123 | 1.00 (0.95-1.06) |  | 288 | 1.00 (0.86-1.16) |
| 5-6 | 78.1 | 4,286 | 1.00 (0.96-1.04) |  | 1,580 | 0.95 (0.89-1.01) |  | 283 | 1.24 (1.07-1.45) |
| 7+ | 99.7 | 5,984 | 1.00 (0.97-1.04) |  | 2,217 | 0.96 (0.91-1.02) |  | 337 | 1.09 (0.94-1.26) |
| Trend per 100 g/d^b^ |  |  | *0.97 (0.93-1.01)* |  |  | *0.95 (0.89-1.02)* |  |  | *0.98 (0.83-1.16)* |
|  |  |  |  |  |  |  |  |  |  |
|  |  | *Χ_1_^2^ for trend = 4.61* | |  | *Χ_1_^2^ for heterogeneity by ER status = 0.45* | | | | |
|  |  |  |  |  |  |  |  |  |  |
| Eggs, portions per week |  |  |  |  |  |  |  |  |  |
| ≤1 | 13.2 | 7,881 | 0.97 (0.94-1.00) |  | 2,982 | 1.01 (0.96-1.06) |  | 462 | 1.05 (0.92-1.18) |
| 2 | 17.2 | 8,507 | 0.98 (0.95-1.00) |  | 3,161 | 0.99 (0.95-1.04) |  | 506 | 1.09 (0.97-1.22) |
| 3 | 22.2 | 5,383 | 1.00 (0.97-1.04) |  | 1,971 | 1.00 (0.94-1.06) |  | 279 | 1.00 (0.86-1.17) |
| 4 | 22 | 3,608 | 1.00 (0.96-1.05) |  | 1,345 | 1.02 (0.95-1.09) |  | 201 | 1.09 (0.91-1.31) |
| 5+ | 29.7 | 2,904 | 0.99 (0.94-1.03) |  | 1,101 | 1.01 (0.93-1.09) |  | 172 | 1.18 (0.96-1.44) |
| *Trend* per *10 g/d*^b^ |  |  | *1.02 (0.99-1.05)* |  |  | *1.00 (0.95-1.06)* |  |  | *1.04 (0.91-1.19)* |
|  |  |  |  |  |  |  |  |  |  |
|  |  | *Χ_1_^2^ for trend = 1.88* | |  | *Χ_1_^2^ for heterogeneity by ER status = 0.18* | | | | |
| Fruit, portions per day^c^ |  |  |  |  |  |  |  |  |  |
| 0-0.99 | 148 | 6,024 | 1.09 (1.05-1.13) |  | 2,264 | 1.11 (1.04-1.17) |  | 354 | 1.20 (1.04-1.39) |
| 1-1.99 | 200 | 9,074 | 1.04 (1.01-1.07) |  | 3,339 | 1.02 (0.97-1.06) |  | 545 | 1.14 (1.03-1.28) |
| 2-2.99 | 251 | 6,564 | 1.00 (0.97-1.03) |  | 2,480 | 1.00 (0.95-1.05) |  | 360 | 1.00 (0.87-1.15) |
| 3-3.99 | 299 | 3,242 | 0.99 (0.94-1.03) |  | 1,220 | 0.98 (0.91-1.05) |  | 188 | 1.04 (0.86-1.26) |
| 4+ | 345 | 2,959 | 0.98 (0.94-1.03) |  | 1,129 | 1.00 (0.92-1.08) |  | 150 | 0.92 (0.74-1.14) |
| Trend per 100 g/d^b^ |  |  | *0.94 (0.92-0.97)* |  |  | *0.95 (0.91-0.99)* |  |  | *0.88 (0.78-0.98)* |
|  |  |  |  |  |  |  |  |  |  |
|  |  | *Χ_1_^2^ for trend = 29.4**** | |  | *Χ_1_^2^ for heterogeneity by ER status = 2.29* | | | | |
|  |  |  |  |  |  |  |  |  |  |

| Vegetables, tablespoons per day |  |  |  |  |  |  |  |  |  | |
| --- | --- | --- | --- | --- | --- | --- | --- | --- | --- | --- |
| <1 | 160 | 2,446 | 1.02 (0.96-1.08) |  | 926 | 1.02 (0.94-1.12) |  | 161 | 1.09 (0.88-1.36) | |
| 1-1.99 | 203 | 7,105 | 1.03 (1.00-1.06) |  | 2,649 | 1.03 (0.98-1.09) |  | 417 | 1.00 (0.87-1.13) | |
| 2-2.99 | 235 | 7,843 | 1.00 (0.97-1.03) |  | 2,882 | 1.00 (0.95-1.05) |  | 460 | 1.00 (0.89-1.13) | |
| 3-3.99 | 258 | 4,108 | 0.94 (0.90-0.98) |  | 1,526 | 0.96 (0.90-1.02) |  | 214 | 0.85 (0.71-1.01) | |
| 4.00+ | 298 | 6,601 | 0.98 (0.94-1.01) |  | 2,524 | 1.02 (0.97-1.08) |  | 349 | 0.91 (0.79-1.06) | |
| Trend per 100 g/d^b^ |  |  | *0.95 (0.91-0.99)* |  |  | *0.99 (0.92-1.05)* |  |  | *0.88 (0.74-1.04)* | |
|  |  |  |  |  |  |  |  |  |  |  |
|  |  | *Χ_1_^2^ for trend = 11.0* | |  | *Χ_1_^2^ for heterogeneity by ER status = 3.38* | | | | | |
|  |  |  |  |  |  |  |  |  |  | |
| Alcohol, drinks/w |  |  |  |  |  |  | |  | |  |
| 0 | 1.6 | 8,608 | 0.97 (0.94-1.00) |  | 3,250 | 1.00 (0.95-1.05) | | 492 | 0.94 (0.83-1.06) | |
| 1-2 | 3.8 | 3,434 | 0.96 (0.92-1.00) |  | 1,317 | 1.00 (0.93-1.07) | | 229 | 1.09 (0.92-1.30) | |
| 3-6 | 9.3 | 5,743 | 1.00 (0.97-1.03) |  | 2,092 | 1.00 (0.95-1.06) | | 337 | 1.00 (0.87-1.15) | |
| 7-14 | 17.9 | 5,859 | 1.05 (1.01-1.08) |  | 2,199 | 1.09 (1.03-1.15) | | 307 | 0.96 (0.83-1.12) | |
| 15+ | 32.1 | 1,867 | 1.23 (1.15-1.30) |  | 690 | 1.28 (1.16-1.42) | | 82 | 1.02 (0.76-1.36) | |
| *Trend per 10 g/d*^b,d^ |  |  | *1.08 (1.05-1.11)* |  |  | *1.09 (1.04-1.13)* | |  | *0.96 (0.86-1.07)* | |
|  |  | *Χ_1_^2^ for trend = 62.1**** | | | *Χ_1_^2^ for heterogeneity by ER status = 1.00* | | | | | |

ER+ve: Estrogen receptor positive breast cancers; ER-ve: Estrogen receptor negative breast cancers; gs-CI: group-specific confidence intervals

****P*<0.0001, after Bonferroni correction

^a^Usual intake taken from the mean of the 24-hour dietary assessments

^b^Trends use the mean of the 24-hour dietary assessments within categories and conventional 99% confidence intervals

^c^Total fruit (fresh, dried and tinned fruit), excluding fruit juice

^d^Trend in alcohol drinkers only

**Table S5.** Associations of sub-types of fruit and fruit juice with breast cancer risk

| **Food or food group** | | | **Usual intake g/d (mean)**^a^ | **Cases** | **RR (99% gs-CI)** |  |
| --- | --- | --- | --- | --- | --- | --- |
| Fresh fruit, portions per day | | |  |  |  |  |
| 0.00-0.99 | | | 140 | 7,764 | 1.00 (0.97-1.03) |  |
| 1.00-1.99 | | | 192 | 9,481 | 0.95 (0.93-0.98) |  |
| 2.00-2.99 | | | 245 | 6,196 | 0.94 (0.91-0.97) |  |
| 3.00-3.99 | | | 288 | 1,972 | 0.92 (0.87-0.98) |  |
| ≥4.00 | | | 338 | 1,399 | 0.91 (0.85-0.98) |  |
| *Trend per 100 g/d*^b^ | | |  |  | *0.95 (0.92-0.98)* |  |
|  |  |  | | | *Χ^2^ for trend = 19.52**** |  |
|  | | |  |  |  |  |
|  | | |  |  |  |  |
| Dried fruit, portions per day | | |  |  |  |  |
| 0.00-0.99 | | | 8.9 | 14,790 | 1.00 (0.98-1.02) |  |
| 1.00-1.99 | | | 17.4 | 1,724 | 0.94 (0.88-1.00) |  |
| 2.00-2.99 | | | 20.6 | 483 | 1.00 (0.89-1.12) |  |
| ≥3.00 | | | 28.9 | 288 | 0.89 (0.77-1.04) |  |
| *Trend per 10 g/d*^b^ | | |  |  | *0.95 (0.90-1.00)* |  |
|  |  |  | | | *Χ^2^ for trend = 7.10** | |
|  | | |  |  |  |  |
|  | | |  |  |  |  |
| Tinned/stewed fruit, portions per day | | |  |  |  |  |
| 0.00-0.99 | | | 14.5 | 17,169 | 1.00 (0.98-1.02) |  |
| 1.00-1.99 | | | 31.5 | 1,026 | 0.94 (0.87-1.02) |  |
| ≥2.00 | | | 37.9 | 279 | 0.95 (0.81-1.11) |  |
| *Trend per 10 g/d*^b^ | | |  |  | *0.97 (0.93-1.01)* |  |
|  |  |  | | | *Χ^2^ for trend = 3.70* |  |
|  | | |  |  |  |  |
|  | | |  |  |  |  |
| Fruit juice, glasses per week | | |  |  |  |  |
| 0.00-0.99 | | | 43.1 | 4,924 | 1.00 (0.96-1.04) |  |
| 1.00-2.99 | | | 63.5 | 3,292 | 1.05 (1.01-1.10) |  |
| 3.00-4.99 | | | 83.4 | 3,628 | 1.01 (0.97-1.06) |  |
| 5.00-6.99 | | | 96.0 | 2,688 | 1.05 (1.00-1.10) |  |
| ≥7.00 | | | 144.5 | 9,354 | 1.03 (1.01-1.06) |  |
| *Trend per 100 g/d*^b^ | | |  |  | *1.02 (0.98-1.06)* |  |
|  |  |  | | | *Χ^2^ for trend = 1.52* |  |

gs-CI: group-specific confidence intervals

**P*<0.01, not corrected for multiple testing

**P*<0.0001, not corrected for multiple testing

^a^Usual intake taken from the mean of the 24-hour dietary assessments

^b^Trends use the mean of the 24-hour dietary assessments within categories and conventional 99% confidence intervals

**Table S6.** Associations of soya intake with breast cancer risk^a^

| **Soya consumption** | **All cases** | |  | **ER+ve cases** | |  | **ER-ve cases** | |
| --- | --- | --- | --- | --- | --- | --- | --- | --- |
|  | **Cases** | **RR (99% CI)** |  | **Cases** | **RR (99% CI)** |  | **Cases** | **RR (99% CI)** |
| Soya meat/tofu at least once per week | 916 | 0.92 (0.84-1.00) |  | 323 | 0.89 (0.77-1.03) |  | 68 | 1.24 (0.90-1.71) |
| Soya milk at least once per week | 882 | 0.92 (0.84-1.01) |  | 343 | 0.94 (0.82-1.09) |  | 57 | 1.03 (0.73-1.46) |
| Soya meat/tofu or soya milk at least once per week | 1,606 | 0.92 (0.86-0.99) |  | 588 | 0.91 (0.81-1.02) |  | 115 | 1.19 (0.92-1.52) |
| Soya meat/tofu and soya milk at least once per week | 192 | 0.87 (0.72-1.05) |  | 78 | 0.94 (0.70-1.26) |  | 10 | 0.80 (0.35-1.81) |

ER+ve: Estrogen receptor positive breast cancers; ER-ve: Estrogen receptor negative breast cancers

^a^RRs are relative to women not in the specified exposure category

**Table S7.** Associations of vegetable types eaten at least once per week with breast cancer risk^a^

| **Vegetable consumption** | **All** | |  | **ER+ve** | |  | **ER-ve** | |
| --- | --- | --- | --- | --- | --- | --- | --- | --- |
|  | **Cases** | **RR (99% CI)** |  | **Cases** | **RR (99% CI)** |  | **Cases** | **RR (99% CI)** |
| **Cruciferous** |  |  |  |  |  |  |  |  |
| Broccoli | 21,940 | 0.97 (0.94-1.01) |  | 8,193 | 0.96 (0.91-1.02) |  | 1,264 | 1.00 (0.86-1.17) |
| Cabbage | 15,358 | 0.98 (0.95-1.01) |  | 5,626 | 0.95 (0.91-1.00) |  | 890 | 1.01 (0.89-1.15) |
| Cauliflower | 17,779 | 0.99 (0.96-1.02) |  | 6,581 | 0.99 (0.94-1.04) |  | 1,013 | 0.98 (0.86-1.12) |
| Brussels sprouts | 10,959 | 1.01 (0.98-1.04) |  | 4,077 | 1.00 (0.95-1.06) |  | 609 | 0.96 (0.84-1.10) |
| Any cruciferous | 27,004 | 0.98 (0.92-1.04) |  | 10,052 | 0.95 (0.86-1.05) |  | 1,542 | 0.97 (0.75-1.25) |
| At least 3 cruciferous | 12,926 | 0.99 (0.96-1.02) |  | 4,778 | 0.98 (0.93-1.03) |  | 734 | 0.98 (0.86-1.11) |
|  |  |  |  |  |  |  |  |  |
| **Allium** |  |  |  |  |  |  |  |  |
| Onions | 22,945 | 0.99 (0.95-1.03) |  | 8,587 | 0.99 (0.93-1.05) |  | 1,299 | 0.97 (0.83-1.14) |
| Garlic | 11,951 | 1.01 (0.97-1.04) |  | 4,417 | 0.98 (0.93-1.04) |  | 630 | 0.92 (0.80-1.05) |
| Leeks | 9,457 | 0.98 (0.95-1.01) |  | 3,672 | 0.99 (0.93-1.04) |  | 519 | 0.90 (0.79-1.04) |
| Any allium | 24,304 | 1.00 (0.96-1.04) |  | 9,077 | 0.98 (0.91-1.05) |  | 1,372 | 0.95 (0.80-1.12) |
| At least 2 allium | 14,849 | 1.00 (0.97-1.03) |  | 5,615 | 1.00 (0.94-1.05) |  | 794 | 0.89 (0.78-1.01) |

ER+ve: Estrogen receptor positive breast cancers; ER-ve: Estrogen receptor negative breast cancers

^a^RRs are relative to women not in the specified exposure category

**Table S8.** Associations of macronutrient intake with breast cancer risk

|  |  |  | | **Cases subdivided by ER status** | | | |
| --- | --- | --- | --- | --- | --- | --- | --- |
|  |  | **All cases** | | **ER+ve** | | **ER-ve** | |
| **Nutrient** | **Usual intake (mean)**^a^ | **Cases** | **RR (99% gs-CI)** | **Cases** | **RR (99% gs-CI)** | **Cases** | **RR (99% gs-CI)** |
|  |  |  |  |  |  |  |  |
| Energy, fifths, kJ |  |  |  |  |  |  |  |
| 1 | 7246 | 5,421 | 0.96 (0.92-0.99) | 2,068 | 0.99 (0.93-1.05) | 325 | 0.99 (0.85-1.15) |
| 2 | 7697 | 5,758 | 0.99 (0.96-1.02) | 2,171 | 1.01 (0.95-1.06) | 374 | 1.12 (0.98-1.28) |
| 3 | 8081 | 5,899 | 1.00 (0.97-1.03) | 2,166 | 1.00 (0.95-1.06) | 330 | 1.00 (0.87-1.15) |
| 4 | 8538 | 5,959 | 1.00 (0.97-1.03) | 2,242 | 1.03 (0.97-1.09) | 318 | 0.97 (0.84-1.13) |
| 5 | 9051 | 5,968 | 0.99 (0.96-1.03) | 2,191 | 1.01 (0.95-1.06) | 311 | 0.96 (0.83-1.11) |
| *Trend per 1000 kJ/d*^b^ |  |  | *1.02 (0.99-1.04)* |  | *1.01 (0.97-1.05)* |  | *0.95 (0.86-1.06)* |
|  |  |  | *Χ_1_^2^ for trend= 2.73* |  | *Χ_1_^2^ for heterogeneity by ER status= 5.22* | | |
|  |  |  |  |  |  |  |  |
| Protein, fifths, % E |  |  |  |  |  |  |  |
| 1 | 14.9 | 5,806 | 0.99 (0.95-1.02) | 2,189 | 0.96 (0.91-1.02) | 351 | 1.02 (0.88-1.17) |
| 2 | 15.8 | 5,702 | 0.96 (0.93-0.99) | 2,096 | 0.92 (0.87-0.97) | 318 | 0.92 (0.80-1.07) |
| 3 | 16.5 | 5,935 | 1.00 (0.97-1.03) | 2,263 | 1.00 (0.95-1.06) | 345 | 1.00 (0.87-1.15) |
| 4 | 16.9 | 5,806 | 0.98 (0.95-1.01) | 2,156 | 0.96 (0.91-1.02) | 326 | 0.95 (0.82-1.09) |
| 5 | 17.8 | 5,756 | 0.98 (0.95-1.02) | 2,134 | 0.96 (0.91-1.02) | 318 | 0.93 (0.80-1.08) |
| *Trend per 1% E*^b^ |  |  | *1.00 (0.99-1.02)* |  | *1.01 (0.98-1.03)* |  | *0.98 (0.91-1.04)* |
|  |  |  |  |  |  | | |
|  |  |  | *Χ_1_^2^ for trend = 0.04* |  | *Χ_1_^2^ for heterogeneity by ER status = 1.53* | | |
|  |  |  |  |  |  |  |  |
| Dairy protein, fifths, % E |  |  |  |  |  |  |  |
| 1 | 2.8 | 5,864 | 0.99 (0.95-1.02) | 2,194 | 0.97 (0.92-1.02) | 324 | 1.02 (0.88-1.18) |
| 2 | 3.3 | 5,899 | 0.99 (0.96-1.02) | 2,185 | 0.97 (0.91-1.02) | 346 | 1.07 (0.93-1.23) |
| 3 | 3.5 | 5,912 | 1.00 (0.97-1.03) | 2,237 | 1.00 (0.95-1.06) | 323 | 1.00 (0.87-1.15) |
| 4 | 3.8 | 5,717 | 0.97 (0.94-1.01) | 2,144 | 0.97 (0.91-1.02) | 336 | 1.03 (0.90-1.19) |
| 5 | 4.1 | 5,613 | 0.97 (0.94-1.01) | 2,078 | 0.96 (0.90-1.01) | 329 | 1.02 (0.88-1.18) |
| *Trend per 1% E*^b^ |  |  | *0.99 (0.96-1.02)* |  | *0.99 (0.94-1.05)* |  | *0.99 (0.86-1.14)* |
|  |  |  |  |  |  | | |
|  |  |  | *Χ_1_^2^ for trend = 0.88* |  | *Χ_1_^2^ for heterogeneity by ER status = 0.00* | | |
|  |  |  |  |  |  |  |  |
| Total fat, fifths, % E |  |  |  |  |  |  |  |
| 1 | 30.4 | 5,703 | 0.98 (0.94-1.01) | 2,130 | 0.98 (0.92-1.04) | 351 | 0.97 (0.84-1.12) |
| 2 | 31.6 | 5,883 | 1.00 (0.97-1.04) | 2,268 | 1.03 (0.98-1.09) | 307 | 0.87 (0.75-1.01) |
| 3 | 32.8 | 5,855 | 1.00 (0.97-1.03) | 2,196 | 1.00 (0.95-1.06) | 346 | 1.00 (0.87-1.15) |
| 4 | 33.7 | 5,751 | 0.98 (0.95-1.02) | 2,119 | 0.96 (0.91-1.02) | 308 | 0.91 (0.78-1.05) |
| 5 | 35.3 | 5,813 | 1.01 (0.97-1.04) | 2,125 | 0.97 (0.92-1.03) | 346 | 1.04 (0.90-1.20) |
| *Trend per 1% E*^b^ |  |  | *1.00 (0.99-1.01)* |  | *0.99 (0.98-1.01)* |  | *1.02 (0.96-1.06)* |
|  |  |  |  |  |  | | |
|  |  |  | *Χ_1_^2^ for trend = 0.94* |  | *Χ_1_^2^ for heterogeneity by ER status = 1.89* | | |
|  |  |  |  |  |  |  |  |
| Saturated fat, fifths, % E |  |  |  |  |  |  |  |
| 1 | 11.3 | 5,636 | 0.97 (0.97-1.01) | 2,112 | 0.98 (0.93-1.04) | 333 | 0.96 (0.83-1.11) |
| 2 | 12.0 | 5,826 | 1.01 (1.01-1.04) | 2,219 | 1.02 (0.97-1.08) | 318 | 0.93 (0.80-1.07) |
| 3 | 12.5 | 5,779 | 1.00 (0.97-1.03) | 2,168 | 1.00 (0.95-1.06) | 339 | 1.00 (0.87-1.15) |
| 4 | 13.3 | 5,934 | 1.03 (0.99-1.06) | 2,208 | 1.02 (0.96-1.07) | 338 | 1.01 (0.88-1.17) |
| 5 | 14.2 | 5,830 | 1.01 (0.98-1.05) | 2,131 | 0.99 (0.94-1.05) | 330 | 1.02 (0.88-1.18) |
| *Trend per 1% E*^b^ |  |  | *1.01 (1.00-1.03)* |  | *1.00 (0.98-1.03)* |  | *1.03 (0.96-1.10)* |
|  |  |  |  |  |  | | |
|  |  |  | *Χ_1_^2^ for trend = 4.50* |  | *Χ_1_^2^ for heterogeneity by ER status = 1.03* | | |
|  |  |  |  |  |  |  |  |
| Carbohydrate, fifths,% E |  |  |  |  |  |  |  |
| 1 | 42.4 | 6,081 | 1.00 (0.96-1.04) | 2,252 | 0.99 (0.93-1.05) | 320 | 0.95 (0.82-1.12) |
| 2 | 46.0 | 5,780 | 0.97 (0.94-1.01) | 2,116 | 0.96 (0.91-1.01) | 318 | 0.92 (0.79-1.06) |
| 3 | 47.4 | 5,875 | 1.00 (0.97-1.03) | 2,182 | 1.00 (0.95-1.06) | 352 | 1.00 (0.87-1.15) |
| 4 | 48.4 | 5,652 | 0.97 (0.94-1.01) | 2,127 | 0.98 (0.93-1.04) | 329 | 0.91 (0.79-1.05) |
| 5 | 50.4 | 5,617 | 0.98 (0.94-1.01) | 2,161 | 1.00 (0.95-1.06) | 339 | 0.92 (0.79-1.06) |
| *Trend per 1% E*^b^ |  |  | *1.00 (0.99-1.00)* |  | *1.00 (0.99-1.01)* |  | *1.00 (0.97-1.02)* |
|  |  |  |  |  |  | | |
|  |  |  | *Χ_1_^2^ for trend = 1.30* |  | *Χ_1_^2^ for heterogeneity by ER status = 0.17* | | |
|  |  |  |  |  |  |  |  |

| Free sugars, fifths, % E |  |  |  |  |  |  |  |
| --- | --- | --- | --- | --- | --- | --- | --- |
| 1 | 8.2 | 5,793 | 0.99 (0.96-1.03) | 2,138 | 0.98 (0.93-1.04) | 335 | 1.01 (0.87-1.17) |
| 2 | 9.4 | 5,765 | 0.98 (0.95-1.02) | 2,128 | 0.97 (0.92-1.03) | 301 | 0.88 (0.76-1.02) |
| 3 | 10.2 | 5,850 | 1.00 (0.97-1.03) | 2,188 | 1.00 (0.95-1.06) | 342 | 1.00 (0.87-1.15) |
| 4 | 11.1 | 5,800 | 1.00 (0.96-1.03) | 2,180 | 1.00 (0.95-1.06) | 333 | 0.97 (0.84-1.12) |
| 5 | 12.8 | 5,797 | 1.01 (0.98-1.05) | 2,204 | 1.03 (0.97-1.09) | 347 | 1.01 (0.88-1.17) |
| *Trend per 1% E*^b^ |  |  | *1.00 (0.99-1.02)* |  | *1.01 (0.99-1.03)* |  | *1.01 (0.97-1.05)* |
|  |  |  |  |  |  | | |
|  |  |  | *Χ_1_^2^ for trend = 1.41* |  | *Χ_1_^2^ for heterogeneity by ER status = 0.30* | | |
|  |  |  |  |  |  |  |  |
| Dietary fiber, fifths, g/d |  |  |  |  |  |  |  |
| 1 | 13.6 | 5,743 | 1.09 (1.04-1.13) | 2,148 | 1.09 (1.01-1.16) | 389 | 1.45 (1.23-1.70) |
| 2 | 15.1 | 5,898 | 1.06 (1.02-1.09) | 2,207 | 1.06 (1.00-1.12) | 322 | 1.12 (0.97-1.29) |
| 3 | 16.1 | 5,723 | 1.00 (0.97-1.03) | 2,124 | 1.00 (0.95-1.06) | 295 | 1.00 (0.86-1.16) |
| 4 | 17.3 | 5,909 | 1.02 (0.98-1.05) | 2,223 | 1.04 (0.98-1.10) | 330 | 1.11 (0.96-1.28) |
| 5 | 19.1 | 5,732 | 0.98 (0.94-1.02) | 2,136 | 0.99 (0.93-1.06) | 322 | 1.10 (0.93-1.29) |
| *Trend per 5 g/d*^b^ |  |  | *0.91 (0.87-0.96)* |  | *0.93 (0.85-1.01)* |  | *0.83 (0.67-1.02)* |
|  |  |  |  |  |  | | |
|  |  |  | *Χ_1_^2^ for trend = 20.5*** |  | *Χ_1_^2^ for heterogeneity by ER status = 1.24* | | |

ER+ve: Estrogen receptor positive breast cancers; ER-ve: Estrogen receptor negative breast cancers; gs-CI: group-specific confidence intervals; E: Energy

***P*<0.001, after Bonferroni correction

^a^Usual intake taken from the mean of the 24-hour dietary assessments

^b^Trends use the mean of the 24-hour dietary assessments within categories and conventional 99% confidence intervals

**Table S9**. Associations of sources of dietary fiber with breast cancer risk

| **Source of fiber (fifths)** | **Mean intake^a^** | **Cases** | **RR (99% gs-CI)** |
| --- | --- | --- | --- |
| Fruit, g/day |  |  |  |
| 1 | 0.7 | 5,961 | 1.00 (0.96-1.04) |
| 2 | 1.6 | 5,839 | 0.97 (0.94-1.00) |
| 3 | 2.5 | 5,826 | 0.94 (0.91-0.97) |
| 4 | 3.7 | 5,757 | 0.93 (0.90-0.96) |
| 5 | 6.5 | 5,622 | 0.91 (0.88-0.95) |
| *Trend per 5 g/d*^b^ |  |  | *0.93 (0.90-0.97)* |
|  |  |  | *Χ^2^ for trend = 20.0**** |
|  |  |  |  |
| Vegetables, g/day |  |  |  |
| 1 | 1.1 | 5,902 | 1.00 (0.96-1.04) |
| 2 | 1.8 | 5,888 | 0.98 (0.95-1.01) |
| 3 | 2.3 | 5,684 | 0.96 (0.93-0.99) |
| 4 | 2.9 | 5,731 | 0.96 (0.92-0.99) |
| 5 | 5.1 | 5,800 | 0.95 (0.92-0.99) |
| *Trend per 5 g/d*^b^ |  |  | *0.95 (0.89-1.01)* |
|  |  |  | *Χ^2^ for trend = 5.32* |
|  |  |  |  |
| Cereals, g/day |  |  |  |
| 1 | 2.2 | 5,820 | 1.00 (0.96-1.04) |
| 2 | 4.0 | 5,794 | 0.98 (0.94-1.01) |
| 3 | 5.5 | 5,737 | 0.95 (0.92-0.98) |
| 4 | 7.1 | 5,795 | 0.95 (0.91-0.98) |
| 5 | 10.1 | 5,859 | 0.95 (0.92-0.99) |
| *Trend per 5 g/d*^b^ |  |  | *0.97 (0.94-1.00)* |
|  |  |  | *Χ^2^ for trend = 5.74* |

gs-CI: group-specific confidence intervals

****P*<0.0001, not corrected for multiple testing

^a^Intake taken from the baseline dietary questionnaire

^b^Trends use the means within categories and conventional 99% confidence intervals

**Table S10.** Associations of alcohol with breast cancer, within categories of fruit and fiber intake

|  | **Alcohol (drinks per week)** | | | | | | | | | |  | **Trend per 10g/d alcohol**^b^ |
| --- | --- | --- | --- | --- | --- | --- | --- | --- | --- | --- | --- | --- |
|  | **0** | | **1-2** | | **3-6** | | **7-14** | | **15+** | |  |  |
|  | **Cases** | **RR (99% gs-CI)** | **Cases** | **RR (99% gs-CI)** | **Cases** | **RR (99% gs-CI)** | **Cases** | **RR (99% gs-CI)** | **Cases** | **RR (99% gs-CI)** |  | **RR (95% CI)** |
|  |  |  |  |  |  |  |  |  |  |  |  |  |
| Fruit, portions per day^a^ | | |  |  |  |  |  |  |  |  |  |  |
| 1 | 1,842 | 1.00 (0.94-1.06) | 610 | 1.04 (0.94-1.16) | 1,036 | 1.05 (0.97-1.14) | 1,150 | 1.14 (1.05-1.23) | 478 | 1.27 (1.13-1.43) |  | 1.07 (1.01-1.13) |
| 2 | 2,690 | 1.00 (0.95-1.06) | 1,084 | 0.94 (0.87-1.02) | 1,832 | 1.00 (0.94-1.06) | 1,886 | 1.05 (0.99-1.12) | 562 | 1.20 (1.07-1.33) |  | 1.08 (1.03-1.13) |
| ≥3 | 3,736 | 0.93 (0.89-0.97) | 1,653 | 0.93 (0.87-0.99) | 2,732 | 0.97 (0.92-1.02) | 2,653 | 0.98 (0.93-1.03) | 736 | 1.17 (1.06-1.29) |  | 1.07 (1.03-1.11) |
|  |  |  |  |  |  |  |  |  |  |  |  | *Χ_2_^2^ =0.14* |
| Fiber, fifths^a^ | |  |  |  |  |  |  |  |  |  |  |  |
| 1 | 1,760 | 1.00 (0.93-1.07) | 531 | 1.03 (0.92-1.16) | 946 | 1.08 (0.99-1.17) | 989 | 1.09 (1.01-1.19) | 414 | 1.32 (1.17-1.51) |  | 1.08 (1.02-1.15) |
| 2 | 1,760 | 1.02 (0.96-1.09) | 647 | 0.96 (0.86-1.06) | 1,135 | 1.03 (0.95-1.11) | 1,280 | 1.15 (1.07-1.23) | 410 | 1.26 (1.11-1.43) |  | 1.10 (1.04-1.16) |
| 3 | 1,684 | 0.98 (0.92-1.05) | 725 | 0.96 (0.87-1.05) | 1,132 | 0.93 (0.86-1.00) | 1,173 | 0.99 (0.92-1.07) | 354 | 1.14 (0.99-1.31) |  | 1.06 (1.00-1.12) |
| 4 | 1,637 | 0.95 (0.89-1.01) | 773 | 0.96 (0.88-1.05) | 1,288 | 1.01 (0.94-1.08) | 1,244 | 1.02 (0.95-1.10) | 371 | 1.23 (1.07-1.41) |  | 1.08 (1.02-1.14) |
| 5 | 1,767 | 0.93 (0.87-0.99) | 758 | 0.92 (0.83-1.01) | 1,242 | 0.98 (0.91-1.05) | 1,173 | 0.98 (0.91-1.06) | 318 | 1.13 (0.98-1.31) |  | 1.06 (1.00-1.12) |
|  |  |  |  |  |  |  |  |  |  |  |  | *Χ_4_^2^ =1.93* |

RRs in categories are relative to the hazard ratio in the lowest intake category of alcohol and either fruit or fiber

^a^Intakes from baseline questionnaire

^b^RR per 10 g/day increase in alcohol, usual intake taken from the mean of the 24-hour dietary assessments
